# Neutralising Antibodies against Ricin Toxin

**DOI:** 10.1371/journal.pone.0020166

**Published:** 2011-05-25

**Authors:** Julie Prigent, Laetitia Panigai, Patricia Lamourette, Didier Sauvaire, Karine Devilliers, Marc Plaisance, Hervé Volland, Christophe Créminon, Stéphanie Simon

**Affiliations:** 1 CEA, iBiTec-S, Service de Pharmacologie et d'Immunoanalyse, CEA Saclay, Gif sur Yvette, France; 2 AFSSAPS, Unité CBR, Montpellier, France; University of Brescia, Italy

## Abstract

The Centers for Disease Control and Prevention have listed the potential bioweapon ricin as a Category B Agent. Ricin is a so-called A/B toxin produced by plants and is one of the deadliest molecules known. It is easy to prepare and no curative treatment is available. An immunotherapeutic approach could be of interest to attenuate or neutralise the effects of the toxin. We sought to characterise neutralising monoclonal antibodies against ricin and to develop an effective therapy. For this purpose, mouse monoclonal antibodies (mAbs) were produced against the two chains of ricin toxin (RTA and RTB). Seven mAbs were selected for their capacity to neutralise the cytotoxic effects of ricin *in vitro*. Three of these, two anti-RTB (RB34 and RB37) and one anti-RTA (RA36), when used in combination improved neutralising capacity *in vitro* with an IC_50_ of 31 ng/ml. Passive administration of association of these three mixed mAbs (4.7 µg) protected mice from intranasal challenges with ricin (5 LD_50_). Among those three antibodies, anti-RTB antibodies protected mice more efficiently than the anti-RTA antibody. The combination of the three antibodies protected mice up to 7.5 hours after ricin challenge. The strong *in vivo* neutralising capacity of this three mAbs combination makes it potentially useful for immunotherapeutic purposes in the case of ricin poisoning or possibly for prevention.

## Introduction

Ricin is a 60–64 kDa glycoprotein of the A–B toxin family, found in the castor bean plant *Ricinus communis* [25]. The toxin consists of two subunits (A and B) linked by a disulfide bridge. The B-chain (RTB) is a galactose-specific lectin which folds into two globular domains, each binding a galactose or N-acetyl galactosamine residue present on glycoproteins and glycolipids at the cell surface [29]. This binding allows ricin to be internalised by endocytosis and retrograde transported to the endoplasmic reticulum where the interchain disulfide bonds are reduced [30]. The A-chain (RTA) is translocated to the cytosol, where its strong *N*-glycosidase activity depurinates an adenine residue of the 28 S ribosomal RNA loop contained within the 60 S subunit [10]. This irreversible process inactivates elongation of polypeptides and leads to cell death.

Because of its high lethality, relative ease of dissemination and availability, the Centers for Disease Control and Prevention consider ricin as a Category B Agent. The symptoms and severity of ricin poisoning depend on the delivery route, the parenteral one being the most toxic [31]. As a bioweapon for terrorism, aerosolised ricin is considered a serious threat and leads to severe lung damage and possibly death. In humans, the estimated lethal dose of ricin is 1–25 µg/kg, administered by injection or inhalation [1], [12]. Currently, no antidote is available for ricin poisoning or prevention [4]. Although several types of therapy are under development, present treatment of possible victims could only be palliative. The literature describes previous attempts to produce vaccines [26], [32], [36], inhibitors of the RTA catalytic activity like chemicals [37], aptamers containing non-natural sugar and purine derivatives [34] and even sugar analogues that prevent binding of ricin to its target [2]. New chemical compounds have been recently described, which inhibit retrograde transport of ricin in the cell, preventing this one to reach its ribosome target [33]. Passive immunisation still remains one of the most effective therapies, immediately active and very specific, allowing the use of low doses of antibodies [3]. In order to prevent ricin poisoning, neutralising antibodies are needed for pre-exposure prophylaxis as well as curative treatment.

In the present study, we produced several murine monoclonal antibodies (mAbs) directed against RTA or RTB and tested them for their neutralising activity against ricin toxin *in vitro*. Seven were active and a combination of three proved most effective: RB34 and RB37 (two anti-RTB mAbs) with RA36 (an anti-RTA mAb). In an *in vivo* mouse protection assay with intranasal challenges of ricin, this combination of three antibodies afforded powerful protection at low concentration. These neutralising mAbs are of great interest for passive immunotherapy for the treatment of ricin poisoning or for pre-exposure prophylaxis.

## Results

### Production of specific mAbs against RTA and RTB

To produce neutralising mAbs against ricin and bypass the natural strong toxicity of this toxin, Balb/c mice were immunised with either the A or the B chain of ricin. However, initial immunisation using 12.5 µg of RTA led to death of the mice, which explains the lower doses of RTA as compared with RTB. Screening of hybridoma supernatants by EIA allowed us to verify the specificity of the antibodies via their binding to A or B chain conjugates. Among a total of 1063 hybridomas from six fusions of spleen cells of mice immunised with RTA, 44 were found to secrete anti-RTA antibodies, and the best 11 clones were selected. A total of 525 hybridomas resulted from the RTB fusion, and 49 clones were found to be positive during screening. Among these, 20 hybridomas were finally selected and stabilised for further investigation. All these different mAbs also recognised the whole toxin, in addition to the separate chain used for their production.

### Monoclonal antibody properties

#### Antibody binding compatibility

A two-site immunometric assay using purified ricin was set up to establish mAb pairs able to bind to the whole toxin simultaneously *in vitro*. All possible combinations of mAb pairs, one for capture and the other biotin-labeled as conjugate, were evaluated (data not shown). A single concentration of ricin was tested (100 ng/ml) in triplicate and compared with nonspecific binding. Selected results obtained with the best mAb conjugates are summarised in Table 1. This allowed us to characterise the mAbs recognising the same (or at least an overlapping) epitope and to define rough specificity groups. Among the antibodies directed against RTB, two (RB34 and RB27) belonged to the same group, while all others (RB13, RB14, RB15, RB24, RB37, RB42 and RB43) were compatible with each other and with these two antibodies. Among the antibodies directed against RTA, RA36 was not compatible with RA30 and RA35, but these two were fully compatible. On the other hand, RA32 and RA33 recognised the same epitopic region and were not compatible. The best results were obtained using mAbs RB14 and RB42 as capture antibodies combined with biotin-labeled RB34, RA35 and RA36 (data not shown).

**Table 1:** Combination assay of antibody binding to ricin.

			Biotin-labeled mAbs							
			Anti-RTB			Anti-RTA				
			RB27	RB34	RB37	RA30	RA31	RA33	RA35	RA36
**Capture mAb**		RB13	**+**	**+**	**+**	**+**	**+**	**+**	**++**	**+**
		RB14	**++**	**++**	**++**	**++**	**++**	**++**	**++**	**++**
		RB15	**+**	**+**	**+**	**+**	**+**	**+**	**+**	**+**
	Anti-RTB	RB24	**+**	**+**	**+**	**+**	**+**	**+**	**++**	**+**
		RB27	**−**	**−**	**++**	**+**	**++**	**+**	**++**	**++**
		RB34	**−**	**−**	**++**	**+**	**++**	**+**	**++**	**++**
		RB37	**++**	**++**	**−**	**++**	**++**	**+**	**++**	**++**
		RB42	**++**	**++**	**+**	**++**	**++**	**++**	**++**	**++**
		RB43	**+**	**+**	**+**	**+**	**+**	**+**	**++**	**+**
		RA28	**+**	**++**	**++**	**+**	**+**	**+**	**++**	**++**
		RA30	**++**	**++**	**++**	**−**	**+**	**++**	**++**	**−**
	Anti-RTA	RA31	**++**	**++**	**++**	**++**	**−**	**++**	**++**	**+**
		RA32	**+**	**++**	**++**	**+**	**+**	**−**	**++**	**++**
		RA33	**+**	**++**	**++**	**++**	**++**	**−**	**++**	**++**
		RA35	**++**	**++**	**++**	**+**	**+**	**++**	**−**	**−**
		RA36	**++**	**++**	**++**	**−**	**+**	**++**	**−**	**−**

A two-site immunometric test was carried out using one capture antibody immobilized on solid phase (1 µg/well) and the other as a biotin-labeled conjugate (100 ng/ml) with ricin at 100 ng/ml. Absorbance was measured after 1 h reaction with Ellman reagent and reported in the table according to the signal intensity: (**−**) <100 mAu; (+) between 100 and 1200 mAu and (++) >1200 mAu.

#### Screening of antibodies in immunoblot

**Figure 1:**
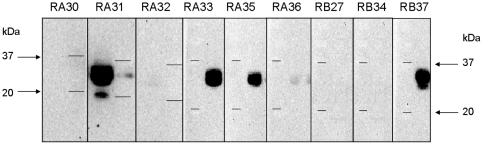
Immunoblot of ricin identified by several anti-ricin antibodies. 2 µg of ricin was migrated in 15% SDS-PAGE and blotted onto PVDF membrane. Primary monoclonal antibodies obtained against the A or B chain were incubated for 1 h to bind to ricin. A secondary antibody HRP-conjugated anti-mouse IgG (diluted 1/2000) was added and proteins were detected after 10 min by chemiluminescence (ECL) using a VersaDoc imaging system (Bio-Rad). Two lanes are shown for each antibody, corresponding to the migration of ricin and of molecular weight markers (two lines, 37 and 20 kDa), respectively.

The 20 anti-RTB and 11 anti-RTA antibodies were tested for their capacity to recognise denatured ricin in reducing conditions in SDS-PAGE/western blotting analysis (Fig. 1). Four out of the 31 antibodies bound the denatured protein and thus possibly a linear epitope during immunoblot experiments (RA31 RA33, RA35 and RB37, Fig. 1).

#### *In vitro* screening of neutralizing mAbs

**Figure 2:**
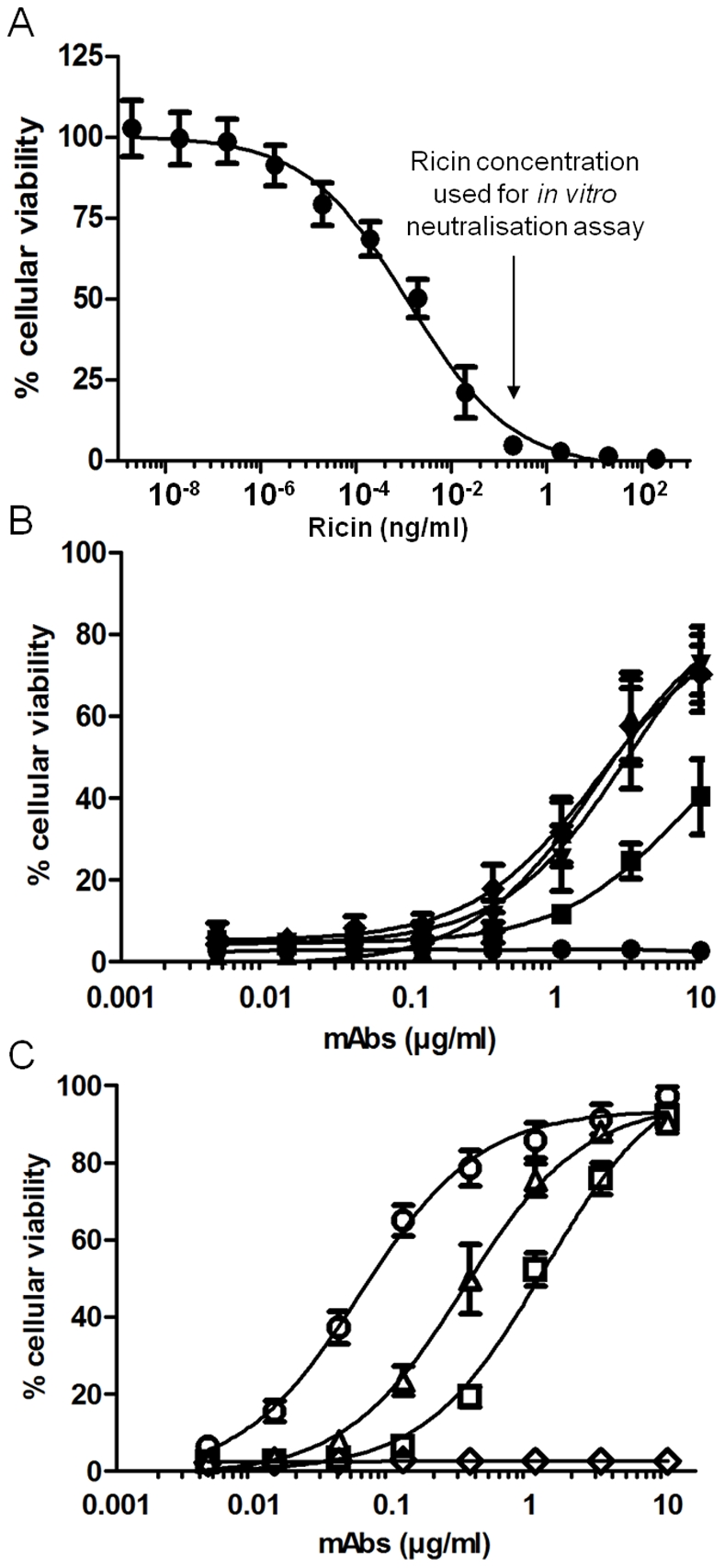
Ricin toxicity and antibody neutralising effect *in vitro* using a viability assay with Jurkat cells. (**A**) Evaluation of ricin toxicity with Jurkat cells. Ricin (0–100 ng/ml) was incubated with 2×10^4^ cells/ml and cell viability was assessed by means of luminescence assay using the Cell titer Glo luminescence kit (Promega). (**B**) Neutralisation assay of ricin using anti-A chain antibodies (RA30: •; RA35: ▪; RA36: ▴; RA32: ▾ and RA33: ♦). (**C**) Neutralisation assay of ricin using anti-B chain antibodies (RB18: ◊; RB27: Δ; RB34: ○ and RB37: □). For Figures (B) and (C), 0.1 ng/ml ricin was pre-incubated with 0–10 µg/ml antibody and then exposed to 2×10^4^ cells/ml for 72 h before assessment of cell viability in the same way as in Figure (A).

All mAbs were tested for their ability to neutralise ricin cytotoxicity in vitro. The ricin concentration necessary to kill more than 95% of Jurkat cells was first determined in a preliminary study (Fig. 2A). A cytotoxic dose that killed 50% of cells (CD_50_) was determined to be 1 pg/ml. A ricin concentration of 0.1 ng/ml was used for antibody screening using 1000 cells per well. The capacity of mAbs to neutralise ricin cytotoxicity was tested using a viability assay. Among the 31 antibodies, seven had a neutralising effect on ricin toxicity (viability greater than 10% at 1 µg/ml), including 4 anti-RTA antibodies, i.e. RA32, RA33, RA35 and RA36, and 3 anti-RTB antibodies, i.e. RB27, RB34 and RB37 (patterns shown in Fig. 2B and 2C, respectively). Non-neutralising antibodies, RA30 and RB18 (representative of all the non-neutralising anti-RTA and anti-RTB antibodies, respectively) are shown as negative controls (less than 5% cell viability at 10 µg/ml). The anti-RTB neutralising mAbs afforded total protection (i.e. 100% cell viability) *in vitro*, whereas this was never achieved even with the highest concentration of anti-RTA mAbs (10 µg/ml, Fig. 2B). Concentrations of neutralising antibodies necessary to maintain 50% cell viability were determined (Table 2). RB34 was the most powerful neutralising mAb, with an IC_50_ of only 58 ng/ml, compared with an IC_50_ at least five (and even 100) times higher for the other mAbs (Table 2).

**Table 2:** Calculated concentration of antibodies that allowed 50% cell viability *in vitro*.

Antibodies	IC_50_ (ng/ml)
RA32 (IgG_1_)	3 173
RA33 (IgG_1_)	1 963
RA35 (IgG_2_b)	>10 000
RA36 (IgG_1_)	1 988
RB27 (IgG_2_b)	333
**RB34** (IgG_1_)	**59**
RB37 (IgG_1_)	1 276
**RB34/RB37**	**41**
RB34/RA36	51
RB27/RB37	201
RB37/RA36	112
RB27/RB37/RA36	190
**RB34/RB37/RA36**	**31**

#### Combination of mAbs to neutralise ricin *in vitro*

**Figure 3:**
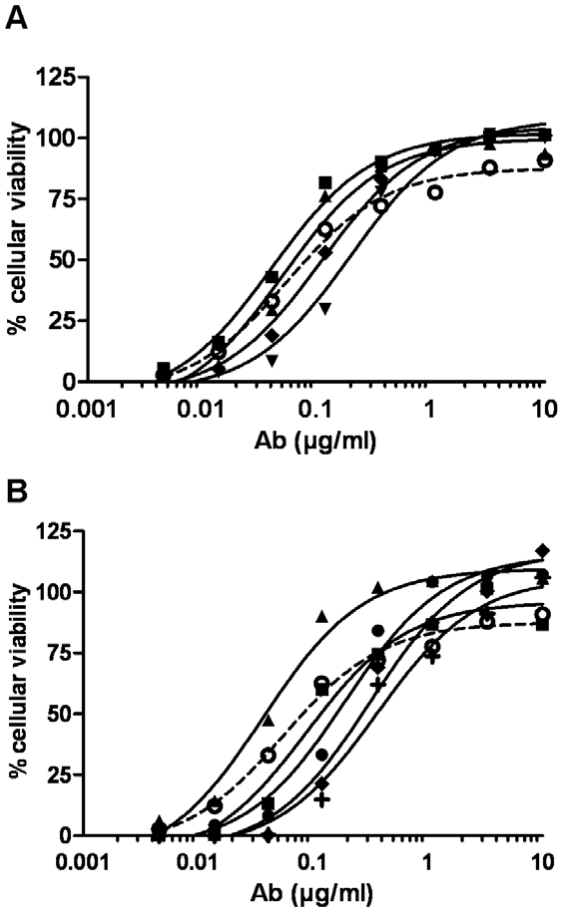
Combination neutralising effect of antibodies against ricin *in vitro*. (**A**) Combination of pairs of antibodies, with RB34 as control (RB34: ○; RB34/RB37: ▴; RB34/RA36: ♦; RB37/RA36: ▾; RB27/RB37: ▪). (**B**) Combination of three or four antibodies, with RB34 as control (RB34: ○; RB27/RB37/RA36: •; RB34/RB37/RA36: ▴; RB34/RB37/RA36/RA32: **+**; RB34/RB37/RA36/RA33: ▪; RB34/RB37/RA36/RA35: ♦). Antibodies were premixed in equimolar ratio at several concentrations (0–10 µg/ml) and incubated with ricin (0.1 ng/ml) before exposure to Jurkat cells. Cell viability was assessed by means of luminescence assay using a Cell titer Glo luminescence kit (Promega).

With a view to increasing ricin neutralisation, protective mAbs were tested in combination. Pairs of antibodies (1∶1 ratio) were evaluated using the same protocol described for screening of the antibodies. As shown in Figure 3A, some mAb pairs showed an additive effect as compared with the mAbs used singly. Best neutralising effects were obtained with pairs including RB34, in particular RB34/RB37 (Fig. 3A), which had an IC_50_ of 41 ng/ml (Table 2). Combination of three and even four mAbs was also tested (Fig. 3B). A slightly greater neutralising effect was observed by adding RA36 to the combination RB34/RB37, with an estimated IC_50_ of 31 ng/ml, which is roughly half that obtained with RB34 alone (Table 2). No additional effect was found by including a fourth mAb (data not shown). RB34 combined with RB37 and RA36 gave cells the greatest protection against ricin *in vitro*. Based on these data, these three antibodies were selected for further characterisation.

### RB34, RB37 and RA36 characterisation

#### Binding kinetics

Kinetic parameters of the three antibodies were measured by Surface Plasmon Resonance biosensor technology using ricin as antigen (Table 3). The dissociation constant, K_D_, was calculated from the ratio of k_off_/k_on_. RB34, RB37 and RA36 exhibit approximately the same K_D_ in the range of 10^−10^ M, partly due to a very small dissociation rate ranging from 3.66 10^−5^ s^−1^ for RB34 to 7.34 10^−5^ s^−1^ for RB37 (Table 3). These very slow k_off_ allow us to estimate a minimum half-life of 2 h, 2.5 h and 3.8 h for the RB37, RA36 and RB34/ricin complexes, respectively. However, these data do not indicate what the half-life would be for the corresponding quaternary complex of ricin and the three-mAb combination.

**Table 3:** Affinity constants of RB34, RB37 and RA36 for ricin.

mAbs	k_off_ (s^−1^) ×10^−5^	k_on_ (M^−1.^s^−1^) ×10^5^	K_D_ (M) ×10^−10^
RB34	3.66±0.27	2.48±0.34	1.50±0.19
RB37	7.34±0.12	3.33±0.39	2.24±0.24
RA36	5.53±0.44	1.58±0.16	3.51±0.46

*K_D_ was calculated from k_on_ and k_off_ with n = 3 for each antibody.

#### Pharmacokinetic studies of RA36, RB34 and RB37 in mice

**Figure 4:**
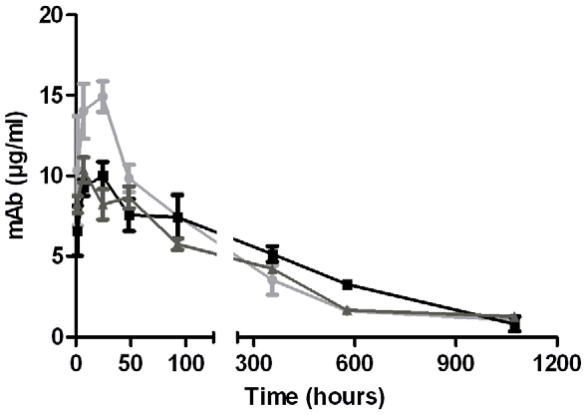
Pharmacokinetic study of RB34, RB37 and RA36 in mice. Purified antibody (50 µg) was injected intraperitoneally into Swiss mice (n = 4). Mice were sacrificed at different times to calculate plasma concentration of mAbs: RB34 (▪); RB37 (▴) and RA36 (•), using an immunoassay.

In order to evaluate the time-window of action of the neutralising antibodies in prophylaxis, it seemed important to determine the half-lives of the different mAbs in mice. After intraperitoneal injection of antibodies in mice, plasma concentrations of RB34, RB37 and RA36 were measured by EIA at different times post-injection. As shown in Figure 4, an initial rapid plasma increase was observed within the first hours after injection for the three antibodies, corresponding to the antibody transfer from the peritoneum to the blood compartment. A peak was reached approximately 18–24 h post-injection, followed by a decrease during the subsequent five weeks. RA36 peaked at 15 µg/ml, whereas RB34 and RB37 were less concentrated (10 µg/ml), a couple of hours after injection. Values when included in a pharmacokinetic (noncompartmental) model allowed evaluating an half-life of 13.2, 14.5 and 17.5 days for RB34, RA36 and RB37, respectively. Then even if the peak plasma concentration was a bit higher for RA36 than for the other mAbs during the first day after injection, the half-lives of these three mAbs are close.

#### Mouse protection assay of anti-ricin antibodies

**Figure 5:**
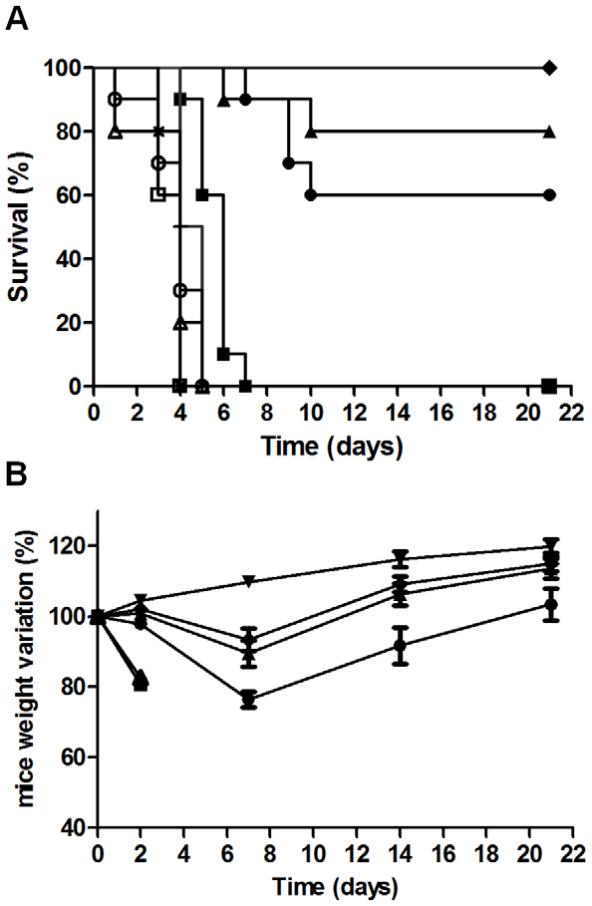
*In vivo* neutralising activity of anti-ricin antibodies combination pre-incubated with ricin (A) Survival curve. CD1 mice were intranasally challenged with 5 LD_50_ of ricin alone (Δ) or pre-incubated with antibodies: several doses of RA36, RB34 and RB37 mixture (Abs) were assessed to obtain antibody/ricin molar ratios of 2 (▪), 5 (•), 10 (▴) and 20 (♦), compared with a nonspecific antibody (ns Ab) in the same concentration (R = 2 (**+**), R = 5 (**X**), R = 10 (○), R = 20 (□)). 50 µl of ricin-antibody complex was administered per mouse and mortality was monitored for 21 days. (**B**) Weight change. In the same experiment, mice were weighed at 0, 2, 7, 14 and 21 days. The percentage of the D0 weight (100%) was calculated, with female CD1 mice as control (▾). Mice injected with ricin alone (Δ) or with mixtures of specific antibodies at R = 2 (▪); R = 5 (•); R = 10 (▴) and R = 20 (♦). The data are representative of two independent experiments.

**Figure 6:**
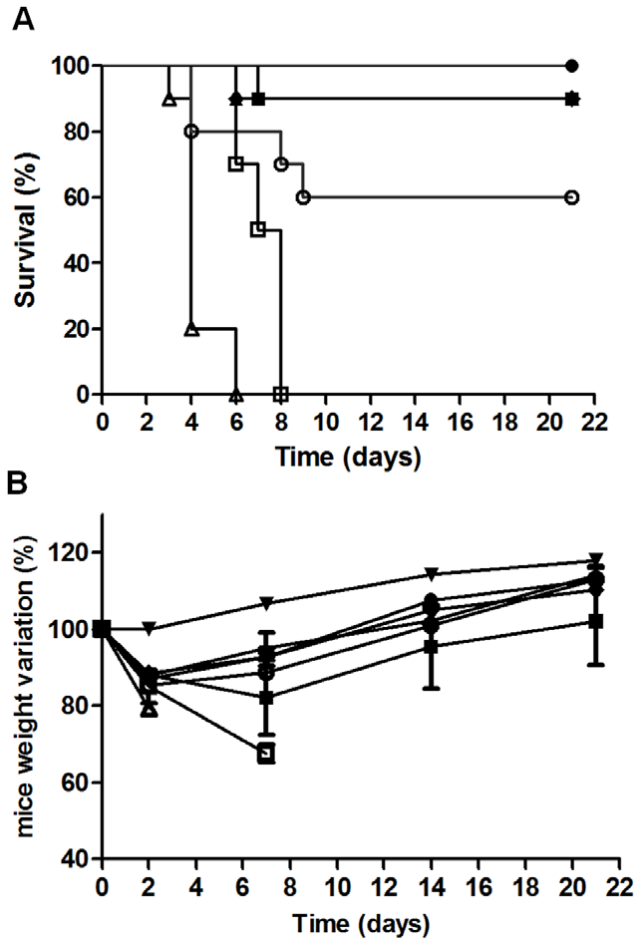
*In vivo* neutralising activity of anti-ricin antibodies combination administered after ricin challenge. (**A**) Survival curve. CD1 mice were intranasally challenged with 5 LD_50_ of ricin alone (Δ) or ricin followed by intravenous injection of 5 mg/kg of antibodies 10 min (▪), 1 h (•), 5 h (♦), 7.5 h (▴), 10 h (○) and 24 h (□) after challenge. (**B**) Weight change. In the same experiment, mice were weighed at 0, 2, 7, 14 and 21 days, taking the weight at day zero as reference (100%), for female CD1 mice as a control (▾), mice injected with ricin (Δ), or ricin followed by intravenous injection of 5 mg/kg of antibodies 10 min (▪), 1 h (•), 5 h (♦), 7.5 h (▴), 10 h (○) and 24 h (□) after challenge. The data are representative of two independent experiments.

**Figure 7:**
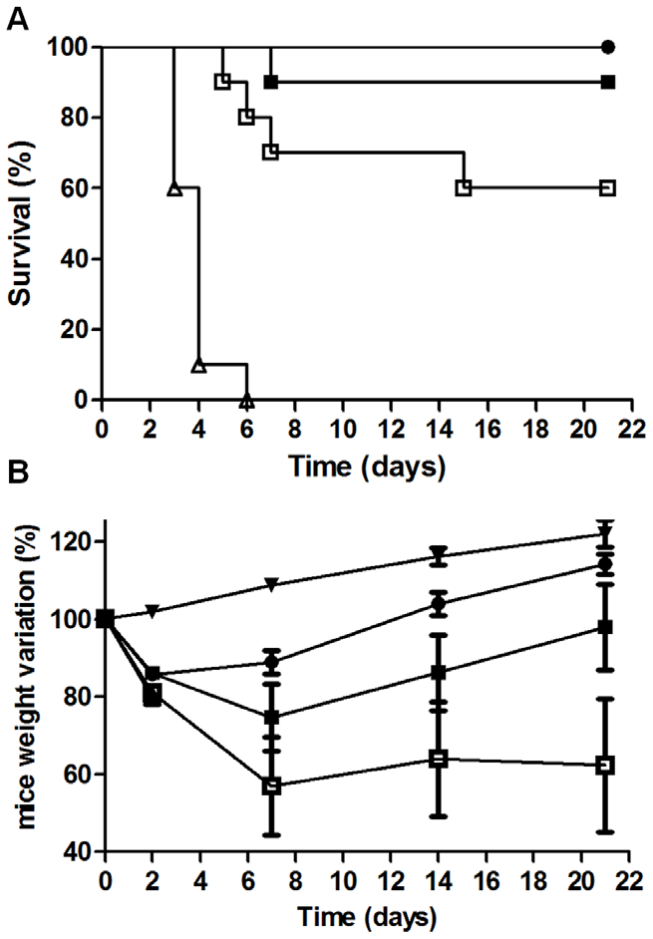
*In vivo* evaluation of each neutralising anti-ricin antibody administered after ricin challenge. (**A**) Survival curve. CD1 mice were intranasally challenged with 5 LD_50_ of ricin alone (Δ) or ricin followed by intravenous injection of 5 mg/kg of RB34 antibody (▪), RB37 antibody (•), or RA36 antibody (□) 1 h after ricin challenge. (**B**) Weight change. In the same experiment, mice were weighed at 0, 2, 7, 14 and 21 days, taking the weight at day zero as reference (100%), for female CD1 mice as a control (▾), mice injected with ricin (Δ), or ricin followed by intravenous injection of 5 mg/kg of mouse weight of RB34 antibody (▪), RB37 antibody (•), or RA36 antibody (□) 1 hour after ricin challenge.

The ability of the RB34, RB37 and RA36 antibodies to neutralise ricin simultaneously was studied using a mouse model for ricin poisoning by intranasal challenge. Ricin was used at 5 LD_50_ (7.5 µg/kg) with several doses of antibodies and survival rate was followed for 21 days (Fig. 5). The antibody combination afforded mice effective protection against ricin poisoning, effectiveness increasing proportionally with the quantity of antibodies. The lowest dose of antibodies (antibody/ricin ratio = 2) did not protect mice, but improved their survival compared with control. With the dose of antibodies corresponding to a ratio 5, a significant protection of mice as compared to the non specific control antibodies was observed (50% viability, fig. 5A). The increase of antibodies concentration (R = 10) allows to reach 100% of protection (Fig. 5A). The analysis of mice weight provided comparable results since the weight loss decreased as the antibody/ricin ratio increased (from −24 to −7%, for a ratio increase from 5 to 20). This mix of antibodies neutralised ricin with great efficiency *in vivo*, and protected the mice against ricin poisoning (5 LD_50_) with a low concentration of antibodies. Combination of antibodies was also tested in a curative protocol, in which 5 LD_50_ of ricin were administered intranasally and antibodies at 5 mg/kg intravenously 10 min, 1 h, 5 h, 7.5 h, 10 h and 24 h after ricin challenge (Figure 6). Antibodies showed a good efficiency to neutralise ricin toxicity up to 7.5 hours after intoxication (Fig. 6A), allowing a 90% mice survival. If mAbs were administered 10 h after intoxication, mice survival falls to 60%, while 24 h after intoxication, antibodies were only able to delay mice death. No visible difference was observed in weight loss between 1 h, 5 h, 7.5 h and 10 h, and weight recovery occurred rapidly (2 days after intoxication). More surprisingly, weight loss was more important and recovery more difficult for the mice injected with antibodies 10 min after ricin challenge (Fig. 6B). In order to evaluate the ability of each antibody to neutralise *in vivo* ricin toxicity, each neutralising antibody was administered intravenously 1 h after intranasal ricin challenge (5DL_50_ of ricin and 5 mg/kg of antibody) (Figure 7). Each of anti-RTB antibodies (RB34 and RB37) proved to be sufficient to neutralise ricin toxin (90% and 100% of mice survival respectively, fig. 7A). Anti-RTA RA36 antibody was less effective to neutralise the toxin with 60% of mice survival. Weight loss appeared the more limited for RB37 antibody and the more important for RA36 (Fig. 7B).

#### Towards the mechanism of action of anti-RTB neutralising antibodies

**Figure 8:**
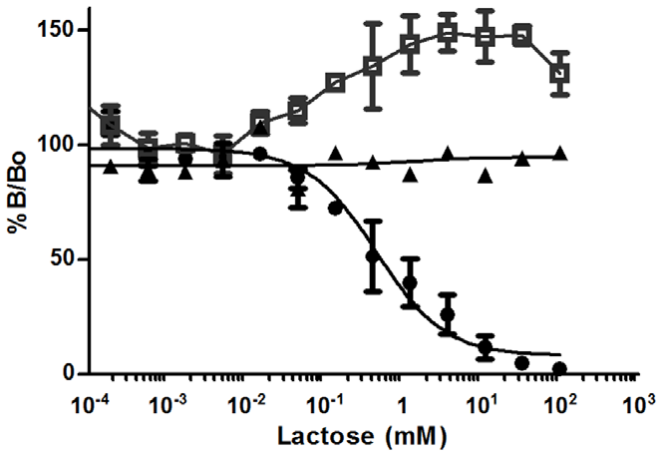
Competition of lactose with antibodies binding to ricin. Neutralising anti-RTB antibodies RB34 (□) and RB37 (•) and a non neutralising antibody (RB18) used as control (▴) were incubated at 75 ng/ml with increasing concentrations of lactose (from 0 to 10 mM) and biotin-labeled RTB (25 ng/ml) in a microtiter plate coated with a polyclonal anti-mouse antibody. Antibodies binding to ricin were further revealed using streptavidin-AChE and Ellman's reagent. Absorbance was measured at 414 nm. B and B_0_ correspond to the absorbance obtained with or without competitor, respectively, allowing to calculate B/B_0_ (expressed as %) for each point.

As anti-RTA antibody was less effective *in vivo* than the anti-RTB ones to inhibit ricin toxicity, preliminary studies were focused on RB34 and RB37 to try to understand their mechanism of action. Indeed, according to the literature, some anti-RTB antibodies could compete with the galactose binding sites of the B-chain to inhibit ricin entry into cells. In order to test this hypothesis for our antibodies, a competition assay using RTB, anti-RTB antibodies and lactose as competitor was set up (Figure 8). Increasing concentrations of lactose (from 0, defining B_0_ i.e 100% of signal, to 100 mM) were used to compete with anti RTB antibodies for binding to ricin. A non neutralising anti-RTB antibody, RB18 was used as a control. RB18 binding to RTB was not modified by lactose. Binding of RB37 to RTB is inhibited by lactose (IC_50_ = 5.2 10^−4^M), suggesting that part of RB37 epitope on ricin toxin corresponds or is close to one of the galactose binding sites. Surprisingly, binding of RB34 to RTB was favoured by increasing concentrations of lactose (up to 150% of B_0_ at 10 mM of lactose). These effects were specific of the galactose moiety of lactose, since no difference of RB34 or RB37 binding was observed when glucose was used as a competitor (data not shown).

## Discussion

The use of neutralising antibodies to bind toxins was first reported in 1894 and is still a treatment of choice, notably for snake bites and scorpion stings. In terms of biosecurity, there is now an increased need to develop vaccines and therapeutic treatments against pathogens and toxins. Ricin is an ubiquitous toxin devoid of drug treatment. In this context, passive immunotherapy is thus of great interest and efficient antibodies should be useful as antidotes both for curative care or for prevention.

We produced several mAbs against RTA and RTB chains of ricin. Among the 11 antibodies produced against RTA, four neutralised ricin toxicity *in vitro*, while three of the 20 anti-RTB antibodies counteracted the cytotoxic effect of ricin *in vitro*. However, it is worth noting that their potential was not identical. Anti-RTB mAbs were more effective than anti-RTA mAbs, and afforded maximum protection (100% cell viability) at a concentration of 3 µg/ml and an IC_50_ close to 60 ng/ml (for the RB34 one). Anti-RTA mAbs were not as potent, and the best RA33 afforded protection of 70% cell viability at highest concentration (10 µg/ml) with an IC_50_ of 1.96 µg/ml (more than 30 times higher than for RB34). Maddaloni *et al.* reported opposite results, with more effective protection using anti-RTA antibodies than anti-RTB antibodies [17], possibly because of the relative affinities of the antibodies for the target. The impact of mAb affinity on protection against toxins has already been studied. Correlation between antibody affinity and serum neutralisation after vaccination has been established for tetanus toxin [7], and verified with the neutralisation of the lethal factor component from *Bacillus anthracis* toxin with high-affinity mAbs [15] and with other neutralising antibodies against anthrax toxins [19]. However, in the present study the different degrees of protection provided by RB34, RB37 and RA36, based on the *in vitro* neutralisation assay, were not related to true differences in affinity for the toxin, as these three antibodies had similar dissociation constants (K_D_) ranging from 0.15 to 0.35 nM. It can thus be hypothesized that the differences in neutralisation *in vitro* are more related to the mechanism of action and epitope specificity of the antibodies than to their kinetic parameters [20]. 

Since we produced various antibodies recognising either RTA or RTB, it was obviously interesting to evaluate and compare different possible combinations to optimise the neutralising capability. For RTA, the situation appears rather complex since RA36 mAb was not compatible with RA30 and RA35, which nevertheless simultaneously bind different epitopes on ricin. This result showed that the epitope recognised by RA36 straddles those of RA30 and RA35. The initial binding compatibility studies demonstrate that starting from nine anti-RTB mAbs, only RB27 and RB34 are mutually exclusive, as they bind to the same epitope on RTB and act in the same mechanism. This was confirmed by *in vitro* neutralisation assay, since the IC_50_ obtained with RB34 alone was greater than the IC_50_ obtained with the pair RB27/RB34 (data not shown), showing that both antibodies compete for toxin binding. As expected, all antibodies from one subgroup were compatible with all antibodies from the other. The *in vitro* neutralising assay showed that the best *in vitro* protection against ricin was provided by the best anti-RTB antibody (RB34) combined with the second best compatible anti-RTB antibody (RB37) and an anti-RTA antibody (RA36), with an IC_50_ of 31 ng/ml. Altogether, these results demonstrate as expected that combining mAbs with different specificities leads to additive or synergistic effects. However, addition of another further antibody did not strengthen the neutralisation potency.

Analysis of studies on botulinum neurotoxin [23], anthrax toxins [16], and more recently SEB toxin [35] shows that the most effective antidotes include several high-affinity and non-cross-reacting mAbs (or Fabs). This was confirmed in the present study, since the results obtained in the mouse protection assay clearly demonstrate that a combination of three antibodies (RB34, RB37 and RA36) provides very good protection against a 5 LD_50_ ricin challenge. Even an antibody/ricin molar ratio of five, i.e. less than two molecules of each of the three antibodies for one molecule of ricin, allows a survival rate of 60%; the rate is 100% with a ratio of 10 (i.e. 4.7 µg of antibodies). The 80% survival observed at a ratio of 20 was not statistically different from the 100% observed at a ratio of 10. It is worth noting that at a ratio of 2, despite 100% mortality, the delay before death was statistically increased. We also evaluated this combination of antibodies in a curative protocol. Mixture of antibodies proved to be efficient to protect mice up to 7.5 h after ricin intoxication, and this delay compares to previous published data for anti RTA antibodies [28]. We then analysed the contribution of each antibody to neutralise ricin *in vivo* and the anti-RTB neutralising antibodies appeared more powerful than the anti-RTA antibody. Moreover, use of RB34 and RB37 alone at 5 mg/kg allowed an almost complete protection against ricin intoxication.

According to the literature, some anti-RTB antibodies might block toxin binding to the cell by interfering with galactose-binding sites [6], [18], [20]. We were thus wondering whether our anti-RTB antibodies could interfere with galactose binding on ricin chain B. Binding of RB34 and RB37 to ricin are influenced by galactose in opposite ways. RB37 binding to ricin is inhibited by galactose and this mAb would thus act by blocking ricin binding to the cell surface through steric hindrance. Conversely, RB34 binding to ricin is enhanced in presence of galactose further suggesting that this antibody would not prevent ricin binding to its target and might act after ricin binding to the cell. The exact epitope recognised by RB34 and the way of inhibiting ricin toxin are currently under investigation.

Mechanism of inhibition of our anti-RTA neutralising antibody is still unknown. It has been described that RTA is subdivided in three folding subdomains, domains 1 and 2 being targets of protective antibodies [24]. In the subdomain 2, peptide 163–174 is sufficient to elicit immunity to ricin [21] and antibody against this peptide partially blocks enzymatic activity. Whether our RA36 antibody would recognise this part of RTA and how it could exert its partial neutralising effect by blocking the catalytic site, or slow down the trafficking process of the toxin by interfering with vesicular retrograde transport or translocation of the RTA across the endoplasmic reticulum [18] remains to be determined.

From the pharmacokinetic studies, the calculated half-lives of the antibodies were close to 15 days, with a peak plasma concentration 5 to 20 h after intraperitoneal injection. These results will be useful for further experiments on prophylaxis in mice. Antibodies could be injected the day before ricin challenge for optimal distribution at the time of cellular exposure to the toxin. With the view to future potential prophylaxis in human subjects, intravenous injection for immediate availability and protection against the toxin will be considered. These different experiments are currently ongoing with the aim of establishing the potential of these mAbs for immunotherapeutic treatment of ricin poisoning.

## Materials and Methods

### Animal experimentation

All experiments were performed in accordance with French and European Community guidelines for laboratory animal handling. The protocols of the *in vivo* neutralisation assays of ricin were approved by the French Health Products Safety Agency (AFSSAPS; protocol no. 2010-CBR-003). All surgery was performed under anesthesia (xylasine/ketamine), and all efforts were made to minimize suffering.

Swiss (CD1) mice (IOPS) used for pharmacokinetic experiments were from Elevage Janvier (Nantes, France) and CD1 (IOPS) mice used for mouse protection assays were from Charles River laboratories (Lyon, France).

### Reagents

Biotin, streptavidin, whole ricin toxin, RTA and RTB were from Sigma. Ricin toxin used for *in vivo* assay was a gift from Dr Beaumelle (CNRS laboratory). Immunoassays were performed with 96-well microtiter plates (Maxisorp, Nunc [Roskilde, Denmark]) and all reagents were diluted in enzyme immunoassay (EIA) buffer (0.1 M phosphate buffer pH 7.4 containing 0.15 M NaCl, 0.1% bovine serum albumin and 0.01% sodium azide). Plates coated with proteins were saturated in EIA buffer (18 h at 4°C) and washed with washing buffer (0.01 M potassium phosphate pH 7.4 containing 0.05% Tween 20).

### Production of monoclonal antibodies against ricin chains A and B

Balb/C mice were immunised once a month for 4 months with either 12.5 µg of RTB or 5 µg of RTA in complete Freund's adjuvant (foot pad injection). Mice were bled two weeks after each immunisation to evaluate and monitor the polyclonal anti-RTA or anti-RTB response in the sera using a specific EIA (see below). The mouse presenting the highest titer was selected for preparation of monoclonal antibodies. Three days before fusion of the spleen cells with the myeloma, the mouse was given a final booster injection (10 µg antigen, i.v. injection). Hybridomas were produced by fusing spleen cells from immunised Balb/c mice with NS1 myeloma cells according to the procedure previously described [13]. Anti-ricin antibodies secreted in culture supernatants were screened using an enzyme immunoassay (EIA, see below). Selected hybridomas were subsequently cloned by limiting dilution and monoclonal antibodies produced in ascites fluids in mice. MAbs were further purified using caprylic acid precipitation [27]. After screening for neutralising properties, the interesting mAbs were purified by affinity chromatography using protein A (ProsepA, Millipore) [11]. Their purity was assessed by polyacrylamide gel electrophoresis (PAGE) in denaturing (SDS) and reducing conditions.

### Enzyme immunoassay

#### Labeling of proteins with biotin

Proteins or mAbs were labeled with biotin and used as conjugates in enzyme immunoassays. 0.67 nmol of antibody or 1.6 nmol of RTA or RTB dissolved in 400 µl of 0.1 M borate buffer pH 9 was incubated with 13.3 nmoles and 32.2 nmoles of biotin-N-hydroxysuccinimide ester (Sigma) respectively dissolved in anhydrous DMF. After 30 min at room temperature (RT), 100 µl of 1 M Tris-HCl pH 8 was added for 1 h at RT. Finally, 500 µl of EIA buffer was added and this preparation was stored frozen at −20°C until use.

#### Evaluation of polyclonal response and screening of mAbs in hybridoma supernatants

Anti-ricin antibodies were detected in sera of immunised mice or hybridoma culture supernatants using EIA. Briefly, 50 µl of serial dilutions of mouse serum in EIA buffer or of each culture supernatant from 96-well culture plates was transferred into microtiter plates coated with goat anti-mouse IgG+IgM antibodies (Jackson Immunoresearch laboratories), before adding 50 µl of biotinylated-RTA or biotinylated-RTB (100 ng/ml). After 18 h reaction at 4°C, plates were washed and 100 µl of AChE-labeled streptavidin conjugate (2 Ellman units [EU]/ml) was added to each well. After 2 h incubation at RT followed by three washing cycles, 200 µl of Ellman's reagent [9] was added and the absorbance was measured at 414 nm after 1 h.

#### Determination of the antibody concentration in mouse plasma

Diluted plasma and purified antibodies used as standard (concentration ranging from 0 to 13.3 ng/ml), were incubated for 18 h at 4°C in 96-microtiter plates coated with whole ricin (100 ng/well). After three washing cycles, 100 µl of 3 EU/ml AChE-labeled goat anti-mouse IgG (conjugate antibody) was added for 2-h reaction. After three washing cycles, 200 µl of Ellman's reagent was added to each well and absorbance was measured at 414 nm after 30 min reaction at RT.

#### Determination of binding complementarity for each pair of mAbs

A combinatorial analysis of mAbs was performed to evaluate their simultaneous binding to whole ricin. A two-site immunometric test was carried out using one antibody immobilized on solid phase for capture and the other as a biotin-labeled conjugate. To determine the “binding complementarity”, experiments were performed by adding 100 µl of ricin (100 ng/ml) and 100 µl of biotin-labeled mAb (100 ng/ml) to the microtiter plate previously coated with one of the mAbs (1 µg/well). After 18 h reaction at 4°C, plates were washed before adding 200 µl/well of AChE-labeled streptavidin conjugate (2 EU/ml) for 1 h reaction at RT. Absorbance at 414 nm was read after 1 h reaction at RT with 200 µl of Ellman's reagent.

#### Competition assay of lactose with anti-RTB antibodies to ricin binding

50 µl of each anti-RTB mAb (75 ng/ml) were added together with 50 µl of biotin-labeled RTB (25 ng/ml) and 50 µl of different concentrations of lactose as competitor (from 0 to 10 mM) in 96 microtiter plates coated with goat anti-mouse IgG. A negative control using glucose as competitor was used.After 18 h reaction at 4°C, plates were washed before adding 200 µl/well of AChE-labeled streptavidin conjugate (2 EU/ml) for 1 h reaction at RT. Absorbance at 414 nm was read after 1 h reaction at RT with 200 µl of Ellman's reagent.. %B/B0 corresponds to the ratio of sample absorbance (B) as compared to the absorbance measured without competitor (B0, corresponding to 100% binding).

#### Western blot analysis

mAbs were tested for their ability to recognise ricin in denaturing and reducing conditions by western blot analysis. Whole ricin was solubilised in Laemmli buffer in reducing conditions (0.25 M Tris-HCl pH 6.8, 4% SDS, 40% glycerol, 0.1% bromophenol blue and 10% β-mercaptoethanol [5]) for 5 min at 95°C. After migration of 2 µg/well of ricin in SDS-PAGE (15% resolving), proteins were blotted onto a PVDF membrane (Amersham Biosciences). Membranes were saturated with PBS containing 0.1% Tween 20, 5% bovine serum albumin and further incubated with the different anti-RTA and RTB mAbs (2 µg/ml) for 1 h at RT. After several washings in PBS containing 0.1% Tween 20, membranes were reacted with a secondary HRP-conjugated anti-mouse IgG (1/2000 [Pierce]) for 20 min at RT. Membranes were washed and proteins bands were detected via chemiluminescence (ECL, Amersham Biosciences) using a VersaDoc imaging system (Bio-Rad).

#### *In vitro* neutralising assays of ricin

The *in vitro* neutralisation of ricin was evaluated using a Jurkat cell viability assay. Jurkat cells (from ATCC) were grown at 37°C with 5% CO_2_, in RPMI 1640 medium supplemented with 10% fetal calf serum, 1% glutamine, 1% sodium pyruvate, 1% penicillin/streptomycin. A standard curve of ricin toxicity was plotted for a ricin concentration range from 0 to 100 ng/ml, using quadruplicate measurements. Neutralising effects of antibodies were tested with a constant ricin concentration of 0.1 ng/ml (i.e. 1.56 pM, the lowest concentration that kills more than 95% of cells). Purified antibodies (concentration ranging from 0 to 10 µg/ml, i.e. 67 nM) were mixed with the toxin in a 50 µl volume and incubated for 30 min at 37°C in 96-well plates. Cells were resuspended at a density of 2×10^4^ cells/ml and 50 µl (1000 cells) was added to the mixture. After 3 days of incubation at 37°C, a luminescence assay with the Cell titer Glo luminescence kit (Promega) was used to measure cell viability. 100% viability was assessed with samples without ricin (cells only), allowing calculation of the percentage viability for each sample. Synergistic effects of neutralising mAbs against ricin were evaluated for combinations of two, three or four mAbs in equal proportions (0 to 67 nM in total), applied to cells in the same conditions as described above.

#### Determination of mAb affinity by surface plasmon resonance

The affinities of neutralising mAbs were determined by surface plasmon resonance (SPR) in a BIAcore2000 instrument (Biacore, Sweden). All analyses were performed at 25°C on a CM5 sensor chip in the running buffer HBS-EP (10 mM Hepes, 150 mM NaCl, 3 mM EDTA and 0,005% surfactant P20, pH 7.4). An anti-mouse IgG was conjugated to the sensor chip using the mouse antibody capture kit (GE Healthcare) according to the manufacturer's instructions. Experiments were performed assuming the existence of a high-affinity antigen/antibody complex [8]. For all neutralising antibodies, kinetic analyses were performed by indirect binding to this CM5 immobilised anti-mouse IgG. The antibody of interest (2 µg/ml) was injected at 5 µl/min for 3 min. After a 10-min stabilisation, whole ricin (concentrations ranging from 0.5 nM to 30 nM) was injected for 3 min at a constant flow rate of 40 µl/min to obtain a maximum signal of 150 resonance units (RU). Dissociation was monitored over a period of 45 min before the chip was regenerated with 10 mM glycine pH 1.7 for 30 s at a flow rate of 50 µl/min. The equilibrium dissociation constant (K_D_) was calculated using the ratio between the dissociation rate constant (k_off_) and the association rate constant (k_on_), as previously described [14], using a Langmuir 1∶1 fit (BIAevaluation Software®, v3.2).

#### Pharmacokinetic analyses of mAbs in mouse

Male CD1 mice 6–8 weeks old (body weight 28 to 32 g) were used for the pharmacokinetic study. 50 µg of 0.22 µm filtered antibodies diluted in PBS buffer was intraperitoneally administered to mice (n = 4). Mice were anaesthetized intraperitoneally with 200 µl of a mixture of xylasine (0.8 mg/ml) and ketamine (9 mg/ml) before blood sampling. Blood was collected 1, 7, 24, 48, 96, 360, 576 and 1032 h after mAb injection and centrifuged for 30 min at 3500 g at 4°C. Plasma was recovered and stored at −20°C until use. Plasma concentration of antibodies was measured by EIA. Pharmacokinetic parameters were determined using the mean antibody concentrations in mice (n = 4) per time point. For calculation of *in vivo* blood clearance, data values were fitted using WinNonlin software (Pharsight).

#### Mouse protection assay of ricin using the combination of three monoclonal antibodies

The ability of a combination of antibodies to protect mice against ricin poisoning was studied *in vivo* using female CD1 mice weighing 22–25 g. Ricin purified from castor beans as previously described [22] was a generous gift of Dr. Beaumelle (CNRS). Ricin LD50 (lethal dose that kills 50% of mice) was evaluated at 1.5 µg/kg using two independent assays in a mouse model with intranasal challenge of ricin (data not shown). Mouse protection was evaluated with a constant ricin concentration of 7.5 µg/kg (5 LD50) and several antibody concentrations (0.9, 2.3, 4.7 and 9.4 µg/mouse corresponding to a molar ratio [mAbs vs ricin] of 2, 5, 10 and 20, respectively N = 10 per experimental group). Antibodies were mixed in equimolar ratio as for *in vitro* assay. Ricin and antibodies were pre-incubated in 50 mM phosphate buffer pH 7.4, 150 mM NaCl and 1 µg/ml gelatin for 1 h at RT before intranasal administration of 50 µl/mouse (n = 10). A negative test was performed with nonspecific antibodies directed against another toxin (anti-botulinum neurotoxin A antibody). Mice were weighed on days 0, 2, 7, 14, and 21 and observed daily until 21 days to plot a survival curve.

#### Therapeutic protection assay against ricin using the combination of three monoclonal antibodies

5 LD50 of ricin was administered intranasally and combination of RA36, RB34 and RB37 antibodies (500 µl, 5 mg/kg, N = 10 mice per group) was injected intravenously at 10 min, 1 h, 5 h, 7.5 h, 10 h and 24 h after ricin administration. Mice were weighed on days 0, 2, 7, 14, and 21 and observed daily until 21 days to plot a survival curve.

#### Evaluation of each neutralising antibody in a mouse protection assay

5 LD50 of ricin was administered intranasally and each of RA36, RB34 and RB37 antibodies (500 µl, 5 mg/kg, N = 10 mice per group) was injected intravenously 1 h after ricin administration. Mice were weighed on days 0, 2, 7, 14, and 21 and observed daily until 21 days to plot a survival curve.
